# Aging and Pattern Complexity Effects on the Visual Span: Evidence from Chinese Character Recognition

**DOI:** 10.3390/vision3010011

**Published:** 2019-03-22

**Authors:** Fang Xie, Lin Li, Sainan Zhao, Jingxin Wang, Kevin B. Paterson, Sarah J. White, Kayleigh L. Warrington

**Affiliations:** 1 Academy of Psychology and Behavior, Tianjin Normal University, Hexi District, Tianjin 300374, China; 2 Department of Neuroscience, Psychology and Behaviour, University of Leicester, University Road, Leicester LE1 9HN, UK

**Keywords:** aging, visual span, Chinese reading, character recognition

## Abstract

Research suggests that pattern complexity (number of strokes) limits the visual span for Chinese characters, and that this may have important consequences for reading. With the present research, we investigated age differences in the visual span for Chinese characters by presenting trigrams of low, medium or high complexity at various locations relative to a central point to young (18–30 years) and older (60+ years) adults. A sentence reading task was used to assess their reading speed. The results showed that span size was smaller for high complexity stimuli compared to low and medium complexity stimuli for both age groups, replicating previous findings with young adult participants. Our results additionally showed that this influence of pattern complexity was greater for the older than younger adults, such that while there was little age difference in span size for low and medium complexity stimuli, span size for high complexity stimuli was almost halved in size for the older compared to the young adults. Finally, our results showed that span size correlated with sentence reading speed, confirming previous findings taken as evidence that the visual span imposes perceptual limits on reading speed. We discuss these findings in relation to age-related difficulty reading Chinese.

## 1. Introduction

Readers move their gaze through text by making rapid eye movements (saccades), separated by brief fixational pauses during which they acquire linguistic information (for a review, [1]). Considerable evidence shows that this eye movement behavior changes with older age, so that older adults (60+ years) read more slowly than young adults (18–30 years) by making more and longer fixations, despite achieving similar levels of comprehension (e.g., [2,3,4,5,6,7,8,9,10,11,12,13,14]). This effect not only is reported widely for alphabetic languages, but recent studies report similar effects for Chinese [15,16,17,18], showing aging effects on eye movement control during reading for both alphabetic and non-alphabetic writing systems.

An important unresolved issue concerns whether this age-related slowdown in reading is due to older adults acquiring less linguistic information on each fixation [8,9,13,19]. Evidence from non-reading tasks indicate that older adults process non-foveal information less effectively than young adults [20,21,22]; and other research indicates that older adulthood is associated with visual declines, especially outside central vision [23,24,25]. Investigations of aging effects on reading often use gaze-contingent paradigms to assess if there are adult age differences in the size of the area of text from which readers can acquire linguistic information on each fixation (the perceptual span; [26,27]). In this paradigm, text is shown normally within a narrow region (window) around gaze, and the window moved in synchrony with the reader’s eye movements so that only a small amount of text is seen normally on each fixation. Typically, these windows are varied in size systematically across an experiment, following the logic that windows which produce normal reading rates must encompass the perceptual span. An influential finding using this method suggests older adults have a smaller and more symmetrical perceptual span compared to young adults ([8]; but see [13]). However, as the paradigm investigates natural reading, performance may be influenced by both attentional and contextual factors associated with reading for comprehension ([28,29,30]; see [31]). For instance, evidence suggests that when a fixated word is more difficult to process, readers allocate less attention to the processing of upcoming words [32,33]. Moreover, readers may also differ in the extent to which they use knowledge about the preceding discourse context to infer the identities of upcoming words, potentially even when upcoming text is degraded so that words are not readily identifiable (see, e.g., [7]). Both factors are likely to influence the apparent size of the perceptual span in a natural reading task. Moreover, these influences are also likely to differ across adult age groups. In particular, older adults typically experience greater difficulty processing the identity of fixated words compared to young adults, and this may limit their allocation of attention to upcoming words. Additionally, other research also suggests that older readers attempt to compensate for this greater difficulty by adopting a more “risky” reading strategy in which they are more likely, compared to young adults, to infer the identities of upcoming words based on contextual knowledge and only partial word information (e.g., [7]). Accordingly, the extent to which adult age differences in the perceptual span during natural reading are a consequence of age-related perceptual limitations as opposed to adult age differences in attentional allocation or language processing during reading remains unclear.

Another approach which is argued to provide a clearer indication of specifically perceptual limitations on the acquisition of linguistic information has used a non-reading task (the trigram task) to estimate the number of letters that can be recognized reliably on each glance without moving the eyes (the visual span; see [31,34,35,36,37,38,39]). In this, trigrams (that do not form a word) are displayed briefly at either a central point or locations to its left and right on each trial in an experiment. Participants are instructed to maintain fixation on the central point while reporting stimuli at each display location, and the visual span is calculated as the number of locations at which stimuli can be reported with at least 80% accuracy. This is widely argued to reflect bottom-up sensory limitations on reading speed, distinct from motor, or linguistic influences associated with natural reading, and thought to derive primarily from effects of visual crowding, which is the inability to recognize a visual object, such as a letter or Chinese character, when it is closely surrounded by similar objects [40,41,42]; see also [43]). Note, however, that research using the trigram task is distinct from research that has used visual memory tasks to assess the capacity of visual short-term memory (e.g., [44]), or visual attention capacity (parameter K in Bundesen’s Theory of Visual Attention model; [45]). Unlike in these tasks, the trigram task does not include a manipulation of the number of stimuli or the interval between stimulus presentation and recall. Instead, the purpose of the trigram task is to assess effects of eccentricity on the recognition of triplets of letters / characters. Moreover, substantial research using this approach has shown that performance on the trigram task is related to reading capabilities. In particular, studies show that visual span size correlates with reading speed both for lists of unrelated words and for normal text (e.g., [36,46]). This research has also demonstrated that increased span size is associated with developmental change in reading speed [47], although these effects have been shown only for English and so it will be important to establish that they generalize cross-linguistically. However, only one study to date has examined adult aging effects on the visual span, using letters as stimuli, and reported smaller visual span sizes as well as slower reading speeds in a separate reading task for older compared to younger adults ([46], see also [48]).

The present study therefore explored this issue further, inspired by a study which used Chinese characters as stimuli and showed that the visual span varies as a function of the pattern complexity of stimuli [49]. Pattern complexity was manipulated in this study by selecting characters that contained varying numbers of strokes (i.e., lines, dashes; and the study also showed that these characters varied in terms of other complexity measures; for further details, see Method section). Even trigrams containing characters with few strokes (lower complexity) produced visual spans smaller than those containing alphabetic letters. But crucially, trigrams containing characters with many strokes (higher complexity) produced smaller spans still, even though all the characters were familiar to participants and commonly used. These findings were attributed to increased visual crowding for characters of greater complexity, which may represent an important and unique source of difficulty in Chinese reading. Importantly, however, as crowding effects appear to be greater for older adults (e.g., [46,50]), as a consequence of ocular and neural changes in older age (see [25]), older readers may have particular difficulty with high complexity characters. To shed light on this issue, we used the same Chinese character stimuli to assess adult age differences in the effects of pattern complexity on the visual span (although, unlike in the study by Wang et al. [49], we did not include alphabetic letter stimuli, as the older adult participants were less familiar with these stimuli and so might produce different visual span effects compared to the younger adults for this reason).

A further concern for the present research was to ensure that task performance by the young and older adults was not affected by fixation inaccuracy. The vast majority of research using the trigram task has relied on instructions to emphasize the importance of fixating a central point prior to stimulus presentation, sometimes supplemented by visual inspection (via a camera) to exclude trials in which participants make anticipatory eye movements (e.g., [34,36,46,49]; but see [37], for a study that used an eye-tracker to ensure fixation accuracy). Substantial evidence indicates instructions alone are insufficient to ensure fixation accuracy in experiments, and that fixation inaccuracy can adversely affect performance in studies assessing the recognition of linguistic stimuli presented each side of a designated fixation location ([51,52]; see also [53,54]). Studies that have directly addressed this issue indicate that fixation control does not appear to differ across adult age groups [55]. However, we used an eye-tracker and a fixation-contingent stimulus presentation procedure to ensure that both age groups of participants in the present experiment accurately fixated the central point before a stimulus was presented. This approach also eliminated the possibility of participants making anticipatory eye movements prior to a stimulus presentation, and it allowed us to objectively identify and exclude trials in which participants made a saccade during the presentation of a stimulus. Taken together, these procedures ensured that our experiment provided an accurate assessment of the perceptibility of character stimuli presented at different locations relative to a central fixation point without contamination by fixation inaccuracy or eye movements.

The experiment enabled us to assess effects of pattern complexity on the visual span for young and older adults. In addition, we used an eye-tracker to assess reading speeds for a set of Chinese sentences. Following Liu et al. [46], we expected older adults to have smaller spans and slower reading speeds than young adults, and for visual span to correlate with reading speed. We also expected to replicate the findings that span size varies with pattern complexity [49]. An especially crucial consideration was whether this influence of pattern complexity was greater for older adults. If so, age differences in span size should be greater for characters with higher rather than lower pattern complexity.

## 2. Materials and Methods

The research (tj-psy 170501) was approved by the research ethics committee in the Academy of Psychology and Behavior at Tianjin Normal University and conducted in accordance with the principles of the Declaration of Helsinki. All participants gave written informed consent.

### 2.1. Participants

Participants were 21 young adults (18–22 years, *M* = 21 years) from Tianjin Normal University and 21 older adults (60–82 years, *M* = 72 years) from the Tianjin community. All were native Mandarin speakers, matched on years of formal education (young adults, *M* = 13 years, range = 11–18 years; older adults, *M* = 12 years, range = 11–15 years, *t*_(40)_ = 0.81, *p* = 0.42, *d* = 0.25). Participants were screened for acuity within the normal range (>20/40 in Snellen values) using a Tumbling E eye chart [56]. The young adults had higher acuity than the older adults (young adults, *M* = 20/20, range = 20/16–20/33; older adults, *M* = 20/29, range = 20/17–20/40, *t*_(40)_ = 5.24, *p* <0.001, *d* = 1.62), as is typical [23]. The older adults were also screened for normal cognitive abilities using the Beijing version of the Montreal Cognitive Assessment (applying a standard exclusion criterion of <26/30, [57]).

### 2.2. Stimuli and Design

Stimuli were 26 low-, 26 medium- and 26 high complexity simplified Chinese characters from Wang et al. [49], selected from the 700 most frequently-used characters in a standard database [58]. A further analysis based on the Modern Chinese corpus [59] showed that these sets of commonly used characters did not differ significantly in terms of their occurrence per million in written usage (low complexity characters, *M* = 1989, SE = 455; medium complexity, *M* = 4031, SE = 1832; high complexity, *M* = 955, SE = 198; *F*_(2,77)_ = 2.04, *p* = 0.137). A key consideration in the original study by Wang et al. [49] was the degree to which the accuracy with which these commonly used characters could be recognized in the trigram task (which was at ceiling for stimuli presented at a central fixation point) and changed as a function of retinal eccentricity. This was also the key consideration in the present research.

Pattern complexity was determined by number and frequency of strokes, ink / pixel density, and perimetric complexity (for further details, see [49]). Characters with an average similarity score below 0.2 and above 0.65 were excluded (low complexity, *M* = 0.30; medium complexity, *M* = 0.42; high complexity, *M* = 0.48). Characters were displayed in Heiti font. At a 60 cm viewing distance, each character subtended 1°, and so exceeded the acuity threshold for character recognition in central vision [60], and were of comparable size to stimuli in previous research.

**Figure 1:**
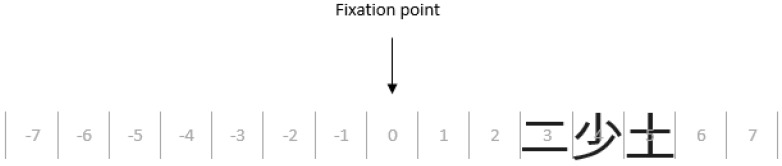
An example of a low complexity trigram presented in a horizontal line at positions 3, 4 and 5. Each trigram (e.g., 二少土) occupies three positions along this horizontal line at varying eccentricities. The trigram is displayed while the participant maintains central fixation. Note that this figure is an illustration of the task only and does not reflect the size or eccentricity used in the experiment.

Each stimulus was a trigram containing characters of the same pattern complexity, with these characters arranged horizontally. An example trial is shown in Figure 1. Trigram members were selected at random from the 26 characters in each complexity set, and did not form words. Each trigram was displayed at either a central point (position 0) or one of 9 positions with increasing eccentricity to the right and left of this point. Characters at adjacent display positions were 1° apart (from the center of one character to the center of the next). Fewer characters were displayed at positions ±8 and ±9 than other positions (only the first character in trigrams at −9 and first and middle characters at −8; and, conversely, only the last character in trigrams at +9 and middle and last characters at +8) and so character-recognition accuracy was assessed only for positions −7 to +7, including position 0. The sets of trigrams of each level of pattern complexity (i.e., low, medium, high) were presented to participants in separate blocks. In each block, trigrams were presented at each location equally often, in randomized order. Each participant viewed 459 trials in total.

The experiment used a mixed design with the between-participants factor age group (young adult, older adult) and within-subjects factors of pattern complexity (low, medium, high) and display location (a central point and 7 locations to the left and right). The dependent variable was reporting accuracy for each display location. Sentence reading speed was assessed separately.

### 2.3. Apparatus and Procedure

An EyeLink 1000 eye-tracker interfaced with a 24 inch high-definition BenQ display screen (1920 × 1080 resolution, 120 Hz refresh rate) monitored participants’ right eye movements (during binocular viewing) and controlled stimulus presentation. Custom software ensured that participants fixated accurately within 0.5° of the central fixation point for at least 100 ms before a stimulus was displayed.

Participants took part individually. At the start of the experiment, each participant was asked to name and indicate the meaning of all the stimuli, which all participants did successfully. The experiment procedure was then explained. Participants were instructed that, on each trial, three characters would be displayed briefly at a central point or one of 9 locations to the right or left, and that they should report these characters (see Figure 1). Participants were additionally instructed that if they were unsure of the identity of a character on any trial, they should provide a guess as a response. The participant was then sat at the eye-tracker and their eye movements were calibrated using a 3-point horizontal procedure. The experiment began with a practice block to familiarize participants with the task and to ensure that they could fixate the central point accurately. Participants then completed 9 blocks of trials (3 for each complexity level, with block order counterbalanced across participants in each age group).

At the beginning of each block, participants were shown the 26 symbols that would be displayed in that block and were encouraged to give responses only from this set. Out of set responses occurred rarely (< 0.1% of trials). At the beginning of each trial, a fixation point (a black dot) appeared at the center of the screen. Once the participant fixated this location for 100 ms, the fixation point disappeared, and a trigram was displayed briefly (for 200 ms). The participant reported the characters they saw in left to right order and an experimenter recorded their response. Recognition accuracy was scored separately for each character in a trigram (1 for a correct response and 0 for an incorrect response) and this score was assigned to that character position. For instance, for a trigram centered at position −3, the left character would appear at position −4 and the right character at position −2. If a participant incorrectly identified the left character in this trigram but correctly identified the center and right character, performance for the trigram would be scored as 0 at position −4 and 1 for positions −3 and −2. Previous research using the trigram task suggests older participants can recognize letter stimuli within 100 ms [48] and so the 200 ms duration used in the current study provides sufficient processing time Durations of around 200 ms are typical of research in this area (see [46,49]). It therefore seemed appropriate to use a 200 ms stimulus presentation duration in the present experiment to ensure comparability with these studies.

To assess reading speed, each participant viewed 20 sentences averaging 21 characters (range = 17–25 characters) in 20-point Song font, presented on the same display screen. Readers’ eye movements were recorded using an EyeLink 1000 eye-tracker. At the viewing distance in the experiment, each character subtended about 1° and so were of normal size for reading. A three-point calibration procedure along the horizontal span covered by each sentence display was performed at the start of the experiment (ensuring spatial accuracy of 0.3° or greater for all participants) and re-calibration performed as necessary. At the beginning of each trial, a fixation square the same size as a character was presented on the left of the screen. Once this was fixated, the square was replaced by the first character of a sentence. Participants then read the sentence for comprehension and pressed a key on the response pad to move to the next trial. Sentence reading times were obtained from the eye movement data and converted into characters per minute reading speeds. For each participant, the entire experiment lasted approximately 45 min.

## 3. Results

We conducted a power analysis to assess the power required to detect an effect of character complexity based on the original Wang et al. [49] study using the Pangea software (https://github.com/jake-westfall/pangea). This indicated that the sample size (*N* = 6) used by Wang et al. was sufficient to detect an effect at 80% power [49]. As the sample size used in the present experiment was substantially larger (*N* = 21 in each age group), it seemed likely our study had sufficient power to detect an effect for both age groups.

### 3.1. Visual Span Size

Reporting accuracy (in %) for each display position (from −7 to +7) was plotted to create a visual-span profile for a given complexity level for each age group. These data can be found in the Supplementary Files. Visual span was calculated by fitting Gaussian curves to these data using MATLAB (version R2017b, The Mathworks Inc., Natick, MA, USA). A single Gaussian and the sum of two Gaussians were fitted individually to the data with the parameters mean, amplitude and standard deviation (resulting in six parameters for the sum of two Gaussians) and the best fit curve selected based on visual inspection and *r^2^* values. Span size was calculated as the width of the fitted curve (number of character positions) at an 80% correct criterion. This measure provides an estimate of the number of character positions encompassed by the visual span by estimating the position at which performance reaches 80% accuracy (note that this threshold is commonly used in visual span studies). As performance for a given position was rarely exactly 80%, the Gaussian fitting estimates the intermediate point at which performance would reach 80% (e.g., if performance accuracy is 90% at one position and 70% at the next, the visual span estimate will fall between the two values). To reflect this, span size is reported to one decimal place.

In addition, to reporting profiles computed by fitting either single Gaussians or a sum of two Gaussians to the data and selecting the best fit based on visual inspection and *r^2^* values, for transparency we also computed profiles based on a single Gaussian. This produced the same pattern of effects, although these fits represent an overestimation of span size compared to raw data, which was not observed when span size was computed using the best-fitting curves produced by either one or two Gaussians. Finally, for completeness, we computed span size as bits of information, using an entropy calculation where information transmitted at a given letter position was computed from the percentage of characters reported accurately. This ranged from 0 bits (for chance accuracy of 3.8% correct) to 4.7 bits (for 100% accuracy; for an explanation of information theory, see [61], and for its application to the visual span, see [37]). This provides a measure of recognition accuracy without the need to assign a criterion value. Chance calculations for character stimuli are less easily defined than for Latin alphabetic stimuli, as characters carry the possibility of an out of set response. We therefore focus discussion on span size as number of characters.

**Figure 2:**
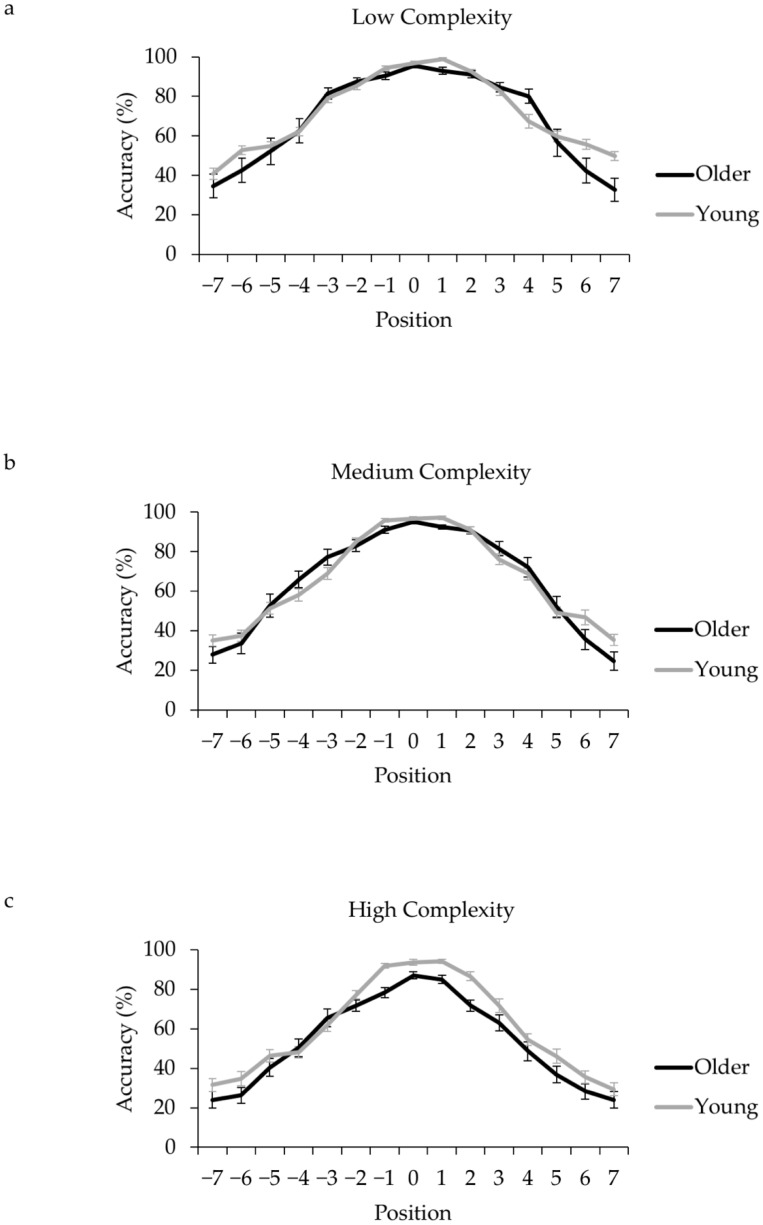
Mean visual span size in characters for (**a**) low complexity stimuli, (**b**) medium complexity stimuli, and (**c**) high complexity stimuli.

Table 1 shows mean visual span (in characters and bits) for each age group and complexity level, and Figure 2 shows mean span profiles. Profiles for each complexity level were similar in shape. In all cases, mean group accuracy was greater than 80% correct at position 0 (central fixation) and declined with increasing distance from this location (however, for high pattern complexity, two older adults did not achieve 80% accuracy even at the center point, discussed in the next section).

Span size for the best-fitting curves was entered into a 2 (age: older adult, young adult) × 3 (complexity: low, medium, high) mixed design Analysis of Variance (ANOVA), and post hoc comparisons performed using Bonferroni corrected pair-wise tests. Two older adults did not achieve 80% accuracy in any position in the high complexity condition, and so a span size of zero was recorded (though note that the pattern of results is identical when these two older adults are excluded). Visual span curves are presented in Figure 2 (Panels a–c). Means and standard errors are summarized in Table 1 and ANOVA statistics are summarized in Table 2, multiple comparison statistics are summarized in Table 3. The ANOVA revealed a main effect of pattern complexity. Span size was smaller for high than medium or low complexity stimuli. Span size was also smaller for medium compared to low complexity stimuli, but not significantly so. This replicates the pattern complexity effects reported by Wang et al. [49].

There was also an interaction between age group and pattern complexity. Follow-up *t*-tests revealed that spans for low complexity and medium complexity stimuli were similar across age groups. However, span size for the high complexity stimuli was smaller for the older adults. Older adults achieved 80% accuracy across about 3 display positions (including the central point), compared to 5.5 positions for young adults. The same pattern of effects was observed for curves computed from a single Gaussian (see Table 2, and note that the Greenhouse-Geisser correction was applied). Moreover, the pattern of pairwise comparisons was the same as reported for analyses based on the best-fitting curves (with no age difference in visual span for low or medium complexity stimuli, *t*s < 0.6, *p*s > 0.5, and a smaller visual span for older compared to young adults for high complexity stimuli, *t*_(40)_ = 3.10, *p* < 0.01). Finally, the pattern of effects was essentially the same for span size calculated as bits of information, with age differences only for high complexity stimuli (*t*_(40)_ = 2.10, *p* < 0.05, *d* = 0.65). Compared to young adults, the older adults appeared to have a smaller span specifically for high complexity stimuli. Further analyses that examined right-left asymmetry in the visual span using an analysis based on the best-fitting curves showed that span size was asymmetrically larger to the right than left of the central point (by 0.4 of a character) across age groups and stimulus complexity, *F*_(1,122)_ = 26.98, *p* < 0.001, η_p_^2^ = 0.18. This asymmetry was also larger for the young compared to older adults (.5 characters to the right for young adults, 0.25 characters to the right for older adults, *F*_(1,122)_ = 26.98, *p* < 0.001, η_p_^2^ = 0.03).

Two older participants achieved less than 80% accuracy for high complexity stimuli at position 0. Accuracy for high complexity stimuli was also overall lower for older than young adults (89% versus 95%, *t*_(40)_ = 2.77, *p* = 0.01, *d* = 0.95). This may be because older adults have greater difficulty recognizing complex characters in central vision. Characters at position 0 were equally likely to be the first, middle or end characters in a trigram. One possibility is that recognition accuracy varied as a function of the location of characters in the trigram. Accuracy at position 0 was therefore assessed as a function of complexity and trigram location. Accuracy was at ceiling (> 90%) for both age groups for low and medium complexity characters at each trigram location. Additionally, accuracy for high complexity stimuli at first and last trigram locations was similar across age groups (first, older adults = 95%, young adults = 96%, *t*_(40)_ =0.79, *p* = 0.437, *d* = 0.24; last, older adults = 91%; young adults = 95%; *t*_(40)_ = 1.58, *p* = 0.12, *d* = 0.49), but lower for older than younger adults at the middle location (older adults = 76%, young adults = 89%, *t*_(40)_ = 3.35, *p* = 0.002, *d* = 1.03). This suggests crowding of middle characters by the first and last characters was greater for the older adults when pattern complexity was high, and that the older adults experienced crowding effects even within central vision. It would be interesting to determine the extent to which the age difference in recognition of the middle character in each trigram can account for the aging effect in our study. This was not possible to assess in the present study, as this would involve probing effects for a small proportion of our data which seemed likely to generate unreliable estimates. Such an approach might nevertheless shed further light on the contribution of crowding effects to adult age differences in the span size.

### 3.2. Sentence Reading Speed

**Figure 3:**
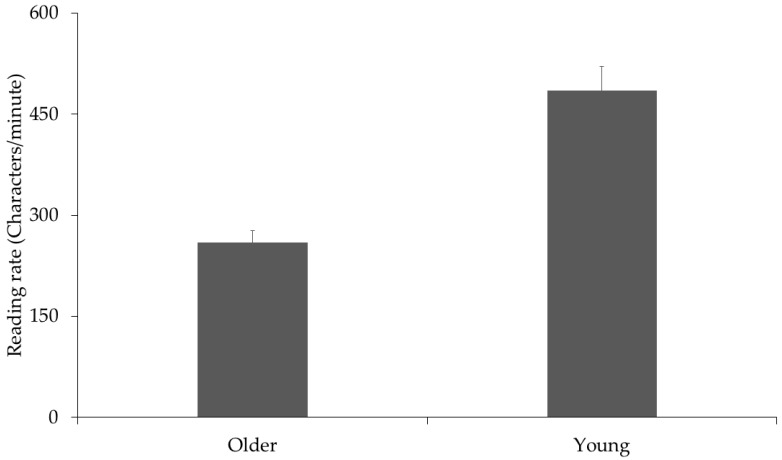
Character per minute reading speeds for young and older adults in the sentence reading task.

Sentence reading speed was calculated as characters per minute reading times for the sentence presentation. These showed that the young adults read more quickly than the older adults (260 vs. 485 characters-per-minute, see Figure 3), replicating the standard finding from eye movement studies showing that older adults read Chinese more slowly [15,16,17,18].

**Figure 4:**
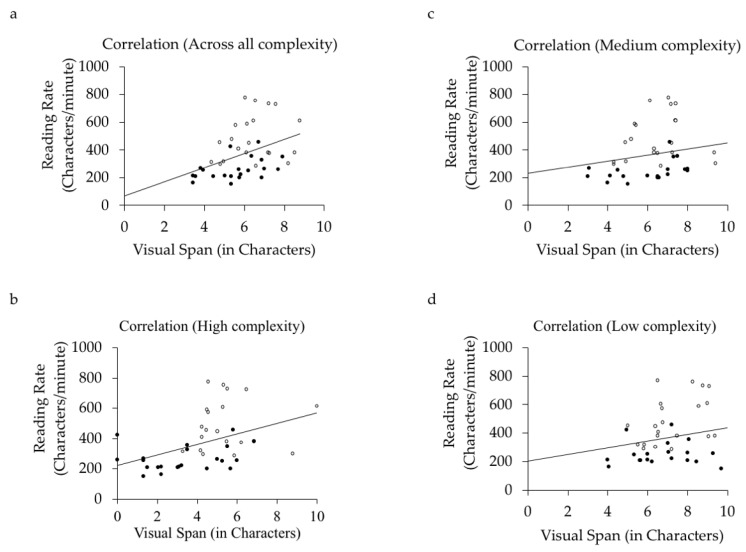
Correlation of reading speed with visual span size, (**a**) collapsed across complexity conditions, and for only (**b**) high complexity, (**c**) medium complexity, and (**d**) low complexity. Filled circles = older adults, unfilled circles = young adults.

We used a Pearson correlation to examine the relationship between visual span size and sentence reading speed, first averaged across all pattern complexity levels and then computed separately for each level of pattern complexity. The results of the correlation analyses are displayed in Figure 4 (Panels a–d).

The analysis for visual spans averaged across all pattern complexity levels showed there was a small but significant positive correlation between span size and reading speed (*r* = 0.24, *p* < 0.01), replicating previous research showing that span size is related to reading speed (e.g., [36,46,47]). However, previous research investigating this issue has examined reading times for alphabetic languages, the present findings show this effect generalizes to non-alphabetic languages like Chinese. Analyses computed separately for each level of pattern complexity showed there was also a positive correlation between span size and reading speed for each level, although this was significant only for the high complexity stimuli (low complexity, *r* = 0.19, *p* = 0.24; medium complexity, *r* = 0.22, *p* = 0.16; high complexity, *r* = 0.43, *p* < 0.01).

## 4. Discussion

We assessed age group differences in pattern complexity effects on the visual span using Chinese characters of varying complexity as stimuli and young and older Chinese adults as participants. Findings showed span size varied as a function of pattern complexity, and was smaller (for both age groups) when stimuli were higher rather than lower in pattern complexity. The shape of the visual-span profiles and span size for the younger participants were similar to previous research [49], and so our experiment clearly elicited typical patterns of performance. While we did not include alphabetic letters as stimuli (as these were less familiar to the older adult participants), previous research shows characters of all complexity, including low complexity characters with only a few strokes, produce visual spans smaller than that for letters. The present findings are in line with this evidence that the visual span for Chinese characters is comparatively small (see also the perceptual span; [62,63]). However, as Chinese characters convey both phonological and semantic information, the smaller span sizes that we observed may be a consequence of the efficiency with which complex linguistic information is transmitted using the Chinese writing system.

It was particularly interesting, however, that we also observed an age difference in pattern complexity effects. Specifically, while there were small (and non-significant) age differences in span size for the lower complexity stimuli, the visual spans for high complexity stimuli were almost halved in size for the older adults compared to the young adults. This suggests that older adults could recognize low complexity characters with ease, but experienced greater difficulty when pattern complexity was high. The only other study to date to investigate aging effects on the visual span, using letter stimuli, reported an approximately 10% shrinkage in span size for older compared to younger adults [46], although visual spans for both age groups were larger than in the present experiment. One possibility is that our participants could recognize characters effectively across a relatively narrow span (no more than 7 character positions) so long as pattern complexity was low, but experienced catastrophic reductions in performance when complexity was high. By comparison, letters stimuli in the study by Liu et al. [46] may have been recognized across a much broader span (up to about 11 letter positions) because of the lower pattern complexity of these stimuli. However, this may also have allowed more subtle visual span effects to emerge due to age differences in peripheral visual processing. What nevertheless seems clear is that pattern complexity has an important role in limiting the visual span, and that this is more pronounced for older adults. Our findings additionally show that age differences in the effects of pattern complexity were not restricted to peripheral vision, and so did not serve only to shrink the visual span, but also impaired character recognition in central vision. Pattern complexity may therefore represent an important and unique source of age-related recognition difficulty that affects both foveal and extrafoveal processing.

Several important questions remain to be addressed, however. The first concerns the precise nature of the effects we observed. Other research that has used the trigram task attributes visual span effects primarily to crowding rather than other perceptual factors ([49]; see also [34,64]); and in the study by Liu et al. [46], the aging effects they observed seemed principally to be due to increased crowding rather than reduced acuity. The age difference in span size for high complexity characters in the present study may also reflect a larger crowding effect for older adults. Consistent with this, we found older adults had particular difficulty recognizing the middle characters of high complexity trigrams in central vision, most likely due to increased crowding by the flanking characters. The effect may be consistent with evidence for crowding in central vision ([65,66], which may be greater for older adults [67]. However, this possible relationship between pattern complexity and crowding remains to be demonstrated more clearly by, for example, comparing adult age differences in span size for isolated characters compared to trigrams to quantify relative effects of acuity and crowding.

It will also be vital to establish if pattern complexity makes an important contribution to age differences in reading performance. Previous research suggests visual span effects reflect bottom-up sensory limitations on reading, distinct from attentional, motor, or linguistic influences associated with natural reading [36]. In the present study, we found older adults read more slowly, and we also observed a correlation between visual span and sentence reading speed. This replicated findings from eye movement studies showing that older adults read Chinese much more slowly than young adults [15,16,17,18]; and also, findings showing a correlation between visual span size and reading speed (e.g., [36,46,47]). However, it will be important to establish more clearly the nature of the relationship between the pattern complexity of linguistic stimuli and reading speed. One possibility is that pattern complexity affects character recognition during reading. Several eye movement studies of Chinese reading already show that young adults have longer fixation times on words that contain characters with higher pattern complexity [18,68,69,70,71]. The nature of the pattern complexity effects in the present experiment may help shed light on the reasons for this effect in eye movements during reading. Moreover, it will be useful to our understanding of aging effects to also determine if older adults produce even larger effects of pattern complexity in fixation times on words in eye movements during natural reading. A further important issue concerns whether pattern complexity serves to limits the number of characters that can be processed on each reading fixation (i.e., the perceptual span), similarly to the effects observed for the visual span in both the present experiment and the study by Wang et al. [49]. Such an effect, especially if larger for older readers, might reveal an important and unique perceptual limitation on the processing of linguistic information that is a particular cause of difficulty for older readers. Moreover, gaining a fuller understanding of pattern complexity effects on both character recognition and the processing of word during natural reading may shed light on unique difficulties faced by Chinese readers, and whether there are important adult age differences in these effects.

In sum, we have reported novel evidence for an adult age difference in the influence of pattern complexity on the number of linguistic stimuli that can be recognized reliably on a single glance without moving the eyes using the trigram task to assess visual span, as well as confirming previous evidence that there is a relationship between span size and reading speed. An important challenge for future research will be to gain a fuller understanding of the underlying causes of adult age differences in these pattern complexity effects, and whether these factors make an important contribution to the difficulty that older adults typically experience during natural reading.

## Figures and Tables

**Table 1:** Means and SE for visual span size (in number of characters and in bits).

	Adult Age Group	High Complexity	Medium Complexity	Low Complexity
Characters (best-fitting curves)	Older	3.1 (0.4)	6.4 (0.5)	7.1 (0.5)
	Young	5.5 (0.4)	6.5 (0.3)	7.2 (0.3)
Characters (single-Gaussian curves)	Older	3.7 (0.6)	6.8 (0.5)	7.6 (0.6)
	Young	5.5 (0.4)	6.5 (0.3)	7.2 (0.3)
Bits	Older	52 (2.1)	58 (2.0)	61 (1.5)
	Young	57 (1.1)	60 (0.7)	62 (0.5)

**Table 2:** ANOVA statistics for visual span size in characters. * refers to the interaction between factors.

		Age Group			Character Complexity			Age Group*Character Complexity		
	df	F	*p*	η_p_^2^	F	*p*	η_p_^2^	F	*p*	η_p_^2^
Visual Span (best-fitting curves)	2, 80	3.91	0.06	0.09	41.26	<0.001	0.51	8.42	<0.001	0.17
Visual Span (single-Gaussian curves)	1.63, 64.59	1.14	0.29	0.03	42.43	<0.001	0.51	5.89	<0.01	0.13

**Table 3:** Multiple comparison statistics examining the interaction between age and complexity.

Character Complexity	*t*	df	*p*	Cohen’s d
Low	0.20	40	0.84	0.07
Medium	0.09	40	0.93	0.03
High	4.40	40	<0.001	1.36

## Supplementary Materials

The dataset supporting these analyses can be found at https://leicester.figshare.com/s/8d820fa0eaccf82b29f0 and the data analysis script can be found at https://leicester.figshare.com/s/61d7083416c7ef42a849.
